# Case Report: Shift from Aggressive Periodontitis to Feline Chronic Gingivostomatitis Is Linked to Increased Microbial Diversity

**DOI:** 10.3390/pathogens14030228

**Published:** 2025-02-26

**Authors:** Claire A. Shaw, Maria Soltero-Rivera, Rodrigo Profeta, Bart C. Weimer

**Affiliations:** 1 Department of Population Health and Reproduction, 100 K Pathogen Genome Project, University of California, Davis, CA 95616, USA; clashaw@ucdavis.edu (C.A.S.); profeta@ucdavis.edu (R.P.); 2 Department of Surgical and Radiological Sciences, University of California, Davis, CA 95616, USA

**Keywords:** aggressive periodontitis, feline chronic gingivostomatitis, oral microbiome, metatranscriptomics, microbial community

## Abstract

Aggressive Periodontitis (AP) and Feline Chronic Gingivostomatitis (FCGS) are two oral inflammatory diseases in cats with unknown etiology. Both conditions present with severe inflammation of the oral cavity and in FCGS it is found with additional deterioration of the non-keratinized mucosa. The oral microbiome is increasingly implicated in disease progression, but little is known about shifts in the microbial community during the AP and FCGS progression. To that end, we used deep metagenomic sequencing with total RNA on three longitudinal samples of the oral microbiome in a cat first diagnosed with AP that progressed to FCGS. This deep sequencing approach revealed that increased diversity at both the genus and species levels marked the shift from AP to FCGS, including increases in *Porphyromonas* and *Treponema* species, and decreased *Streptobacillus* species. The metatranscriptomes were then probed for expression of antimicrobial resistance genes and virulence factors. Disease-related genes that include *cheY*, and *ompP5* were expressed in early AP and FCGS, while others like *galU* were only expressed in one or the other disease state. Both genus and species-level shifts were observed along the longitudinal microbiome samples with a noted increase in species diversity in the FCGS-associated microbiome. Corroborating that functional shifts accompany taxonomic changes, the AMR and virulence factor expression similarly changed between the sampling points. Together, these taxonomic and functional shifts indicate that AP and FCGS are potentially linked and may be marked by changes in the oral microbiome, which supports the development of microbial-based clinical diagnostics and therapeutics.

## 1. Introduction

Aggressive periodontitis (AP) and Feline Chronic Gingivostomatitis (FCGS) are two debilitating diseases of the cat’s oral cavity marked by progressing inflammation and deterioration leading to early tooth loss [1]. Both diseases decrease the quality of life and are prevalent, with AP affecting up to 13% of juvenile cats and FCGS affecting up to 26% of adult cats [1,2]. While clinically AP and FCGS are not considered to be connected diseases, a portion of cats diagnosed with AP in early life have been later diagnosed with FCGS [1]. Both chronic diseases are important contributors to declining oral health and subsequent sequelae, but the origin of either disease remains unknown. Ongoing work supports a multifactorial cause that includes a combination of host immune status, systemic infection, and changes in the oral microbiota [3,4,5,6,7,8,9]. The lack of a known cause for either AP or FCGS has led to limited treatment options for clinicians that largely include near full-mouth or full-mouth tooth extractions, and medical management including pain control and immunosuppressive or modulating therapies that are often times given lifelong [10]. A deeper understanding of the suspected contributing factors, like related shifts in the oral microbiome, is therefore necessary to improve treatment options and patient outcomes. 

The oral microbiome is a known contributor to oral health and disease in cats and other mammals [11,12,13]. Though there is some variation between individuals, the healthy oral microbiota in cats is dominated by members of *Actinobacteria*, *Bacteroidetes*, *Firmicutes*, and *Proteobacteria* [14,15]. These microbes colonize the hard dental structures and soft tissue of the oral cavity and together help digest food particles, influence the immune responses of the host, and contribute to systemic health [16,17,18]. When this delicate compositional and functional balance is broken through antibiotic treatment, dietary changes, infection, or other influences, the host becomes susceptible to progressive oral dysfunction and disease [19,20,21]. Periodontal pathogens also contribute to the development and exacerbation of dysbiosis and disease [22,23,24,25]. While no single pathobiont has been identified for AP or for FCGS in cats, periodontal pathogens like *Aggregatibacter actinomycetemcomitans*, *Porphymonas gingivalis*, and *Treponema denticola* have previously been associated with AP in humans [26,27]. These organisms may well play a role in cat oral health as well [15]. The association of dental pathogens with some periodontal diseases and the known contributions of the oral microbiome to maintaining host health together underscore how the oral microbiome contributes to both disease and health.

Though the importance of the microbiome in driving disease is established, the underlying triggers driving the switch from a commensal organism to an antagonistic pathobiont remain unknown [28]. Given the importance of the oral microbiome in either initiating or driving the progression of AP and FGCS, characterizing community shifts over time is a necessary step towards developing a mechanistic understanding of the oral microbiome’s contribution to dental health. To that end, we used deep sequencing of total RNA from buccal swabs in a cat first diagnosed with AP and later diagnosed with FCGS. The longitudinal sampling of three time points during the progression of dental disease coupled with the deep sequencing approach provides a previously unparalleled look at the shifting oral microbiome in two understudied cat oral diseases. Taken together, these microbiome snapshots across time chronicle an oral microbiome in flux and highlight that the microbial composition in the mouth changes over taxonomic levels with progressive inflammation and worsening oral lesions. Notably, the use of total RNA sequencing in this work illustrates changing microbial activity across time, via functional profiling and enrichment analyses of activities that include antimicrobial resistance (AMR) genes and virulence factors. While this work focuses on a single cat and thus requires larger studies to validate the findings at a population level, the observations here of an actively changing oral microbiome longitudinally support the hypothesis that the oral microbiome may function as a marker for and possibly a contributor to disease status.

## 2. Case Description

A 7-month-old female spayed domestic short hair weighing 3.9 kg presented 4 months after being rescued from a shelter in Northern CA with suspected juvenile gingivitis. About three weeks prior to presentation, the primary care veterinarian had noted enlarged mandibular lymph nodes, moderate gingivitis with buccal mucositis, and halitosis. Feline leukemia virus (FeLV) and feline calicivirus (FIV) testing were negative at that time. No evidence of oral discomfort was noted by the owner.

On presentation to a board-certified veterinary dentist, mild to moderate gingival enlargement with moderate-to-severe gingivitis was noted on the maxillary and mandibular premolar and molar teeth. Mild inflammation of the buccal mucosa was noted, and the mandibular lymph nodes were moderately enlarged and firm. The rest of the physical examination was unremarkable. The pre-operative blood minimum database was unremarkable.

Anesthetized evaluation two weeks later including dental charting and intraoral dental radiographs was performed and revealed pseudo pocketing on the maxillary and mandibular premolar and molar teeth and stage 2 periodontal disease. Gingival recontouring and a periodontal treatment were performed. Extractions were not indicated at that time. A biopsy of the inflamed gingiva showed severe, chronic, erosive to proliferative lymphoplasmacytic and neutrophilic gingivitis.

The patient healed from gingivoplasty uneventfully; however, inflammation of the caudal mucosa continued to worsen. Re-evaluation 3 months after surgery showed persistent gingivitis of the premolar and molar teeth and worsening of the caudal oral cavity despite treatment with moderate inflammation and ulceration noted. The incisors and canine teeth were spared. The patient had gained weight and the body condition score at the time was 7/9 (4.5 kg). Given the progression of inflammation and no response to conservative management, FCGS was discussed and partial mouth extractions were recommended and performed five months after the initial presentation. A biopsy of lesional tissue from the caudal oral mucosa then showed marked multifocal to coalescing chronic neutrophilic and plasmacytic inflammation with intralesional bacterial colonies.

Extractions healed uneventfully; persistent but mild inflammation was seen two months post-operatively and then the patient was lost to follow-up until two years later. Anesthetized evaluation then showed stage 2 periodontal disease in the remaining incisors early stage 3 (30% attachment loss) in the remaining canine teeth and persistent mild inflammation in the caudal oral cavity. A great appetite, energy level, and persistent over-conditioning (BCS 7/9 at 5.2 kg) were noted during that visit. No plaque and calculus control was ever conducted at home nor was this patient treated with immunosuppressives. Onsior was prescribed after the first biopsy (6 mg PO QD for 2 days). Amoxicillin clavulanate was prescribed after the extraction of premolar and molar teeth (62.5 mg PO BID for 10 days). Pain management was otherwise accomplished with buprenorphine (0.15 mg PO up to TID) and gabapentin (25–50 mg PO up to TID) as needed.

**Figure 1:**
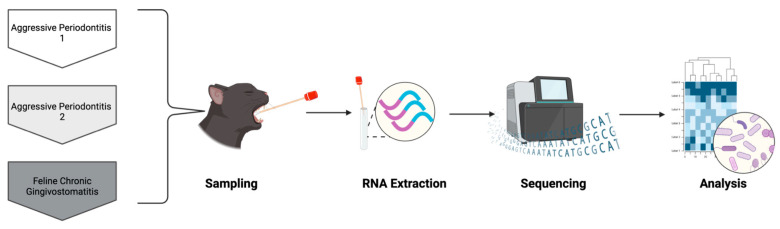
Schematic overview of the extraction and processing of three oral swabs.

The collection and study design were reviewed and approved by the University of California-Davis Institutional Animal Care and Use Committee (IACUC #22738) and signed owner consent was obtained before sampling. A swab of the caudal buccal mucosa was taken during each of three visits (13 January 2021, 10 February 2021, 26 May 2022) and the same method for extraction and sequencing was applied to all three swabs (Figure 1) and as described previously [8].

A cytobrush (FLOQSwabs, Coplan, Italy, EU) was used to swab the oral mucosa lateral to the palatoglossal folds, then placed in 500 μL of DNA/RNA Shield (Zymo, Irvine, CA, USA), vortexed, and stored at −20 °C. Bacterial cells were enzymatically lysed according to the protocol used by the 100 K pathogen project [29], and then RNA was isolated using Trizol LS (Ambion, Austin, TX, USA) according to manufacturer instructions. RNA sequencing libraries were prepared as described previously [30,31,32], with RNA purity and integrity confirmed using TapeStation (Agilent Technologies Inc., Santa Clara, CA, USA). Sequencing libraries were constructed using the enzymatic-based KAPA HyperPlus Library Preparation kit (KK8514) (Kappa Biosystems, Wilmington, MA, USA) on a PerkinElmer Sciclone G3 (PerkinElmer Inc. Waltham, MA, USA) and sequenced on an Illumina NovaSeq S4 (Illumina, San Diego, CA, USA).

Trimmomatic (version 0.39) [33] was first used to remove low-quality sequences and sequencing adapters; then, sequence data quality was reviewed with FastQC (version 0.11.9) [34]. Kraken2 with a microbial reference database, using standard settings (k-mer size = 35), was used to assign taxonomy, and Bracken (version 2.6.1) [35] was then used to estimate the relative proportion of respective taxa at the species level [30]. Expression of AMR genes was determined by running Trinity (v2.15.1) [36] assembled reads through the Comprehensive Antimicrobial Resistance Database (CARD, built 10 August 2023) [37]. Virulence factor expression was evaluated using the Virulence Factor Database (VFDB, built on 10 August 2023) [38]. The STRING database (accessed on 2 October 2024) was used to search for gene and protein connections between AMR and virulence genes in selected oral microorganisms [39]. Shannon diversity and Bray-Curtis dissimilarity were calculated using the diversity function of the vegan package (Version 2.6-8) in R (Version 4.4.1), and subsequently plotted using Prism 10 (GraphPad, Menlo Park, CA, USA). The correlation plot was made using R (Version 4.2.3) in tandem with Inkscape (Version 1.0) and accessed via GitHub accessed on 1 May 2020. (https://github.com/inkscape/inkscape). The Venn diagram and alluvial plot were made using ggplot (Version 3.5.1) in R (Version 4.4.1), with Adobe Illustrator (Version 28.7.5) (Adobe, San Jose, CA, USA) used to reformat text placement and size. All other figures were made using BioRender (biorender.com).

## 3. Results

**Figure 2:**
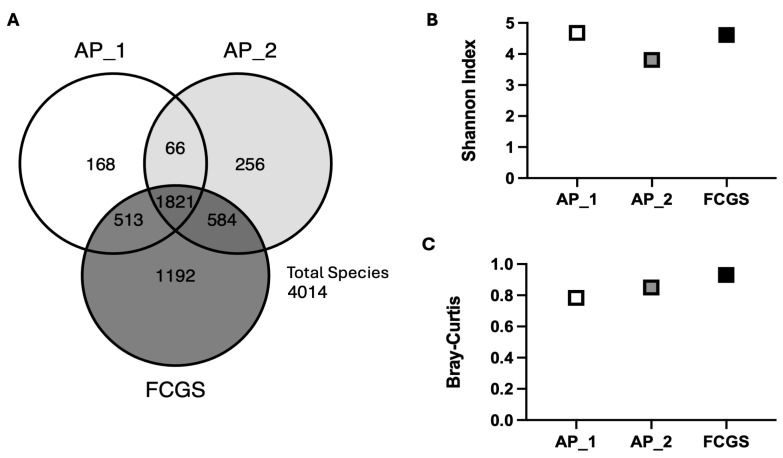
Species-level diversity increases from early aggressive periodontitis (AP_1 and AP_2) through severe dental disease (FCGS). (**A**) Venn diagram illustrating the unique number of species and number of shared species found in each sampling point. (**B**) Alpha diversity, as indicated by the Shannon Diversity Index, at each sampling point, and (**C**) beta diversity illustrated by Bray-Curtis. Across all panels light gray represents the first AP sample, medium gray represents the second AP sample and dark gray represents the FCGS-associated sample.

The species-level diversity in the oral microbiome of a single cat with progressive dental disease increased from early AP to the onset of FCGS (Figure 2). A total of 4014 microbial species were found among all three sampling points. The number of species unique to each oral microbiome increased from 168 in the first AP sample, to 256 in the second AP sample and 1192 in the FCGS sample (Figure 2A). Notably, AP_1 and AP_2 samples shared 513 and 584 species with FCGS, respectively, while only sharing 66 species with each other. The increased number of unique species in the most progressed disease state suggests species-level remodeling of the oral microbial community underlies disease status as opposed to the outgrowth of a pathobiont or collapse of the community. Further supporting this observation, all three oral samples had high α- and β-diversity indices (Figure 2A,B). The high Shannon Diversity Index for all three microbiome samples suggests notable diversity in each microbiome and an even distribution of species abundance (AP_1 = 4.7, AP_2 = 3.8, FCGS = 4.6). Reflecting the observed increase in oral microbial diversity from inflammation limited to the periodontium (AP) to more generalized inflammation affecting mucosa in addition (FCGS), the Bray-Curtis dissimilarity index for all three samples was close to one and consistently increased from AP_1 (0.78) to AP_2 (0.85) to FCGS (0.93). Together, the number of unique species in each sampling point and the general trend of increasing α- and β-diversity along the disease continuum from focal to diffuse inflammation crossing over the mucogingival line supports that microbiome remodeling may be an important facet in determining disease status.

**Figure 3:**
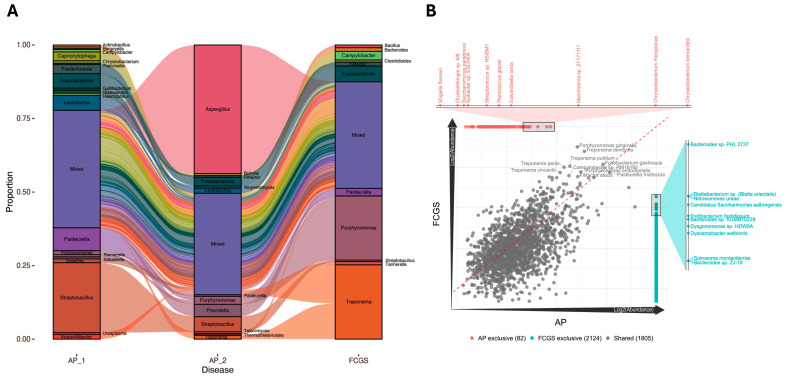
The oral microbiome shows compositional changes across time and disease status at both the genus and species levels. (**A**) Alluvial plot illustrating changes in microbial proportions with the bars indicating genus-level composition and the alluvia in-between displaying the dynamic shifts between each sampling point. The ‘Mixed’ box contains any genus with less than 1% total proportion. (**B**) Correlation plot showing the overlapping and unique species by disease, with AP_1 and AP_2 aggregated into a single community profile. Each dot represents an individual species with the centerline indicating equal proportion in each group.

The observed increase in microbial diversity at the species levels from early AP to later FCGS suggests that these inflammatory diseases are not the result of a single pathobiont and instead are likely to be initiated or exacerbated by complex community dynamics in the oral microbiome that shift over time. To better understand the dynamic changes across the disease continuum, each oral microbiome composition was assessed at both the genus and species level and then compared across time and disease classification (Figure 3). A comparison of the microbial composition in a time series revealed clear changes in the genera between the AP_1 and AP_2 samples, as well as between the second AP sample and FCGS (Figure 3A). While the oral microbiome of AP_1 contained 19 genera with total proportions above 1% each, the second AP microbiome sampled contains 13 genera with *Aspergillus* accounting for almost half of the community proportion. This increased *Aspergillus* population is concomitant with a decrease in the proportion of multiple genera including *Capnocytophaga*, *Frederiksenia*, *Pasteurella*, and *Streptobacillus*. Intriguingly, the microbial community shifts at the genus level once again from AP_2 to FCGS. In the FCGS community, there was a slightly more even distribution, reflected by the high Shannon Index of 4.6, that included *Campylobacter*, *Fusobacterium*, *Pasteurella*, *Porphymonas*, and *Treponema*. The pattern of a diverse oral microbiome in AP_1_, to a markedly different composition to AP_2_, to a completely different composition yet once again an evenly distributed one in FCGS raises questions as to whether changes in specific species are connected to disease presentation and progressive tissue damage.

To evaluate the importance of specific species in the two disease presentations, AP and FCGS, the two AP samples were aggregated and compared to the FCGS microbiome in a correlation plot (Figure 3B). Multiple microbes were shared in approximately equal proportion across the two disease microbiomes including *Porphyromonas gingivalis*, *Treponema denticola*, *Pasteurella multocida*, and *Fusobacterium gastrosuis*. The relatively dense cloud of 1805 points clustered around the center diagonal line suggests notable overlap in species present in the AP and FCGS microbiomes and that the proportions of these species are similar between the two diseases.

**Figure 4:**
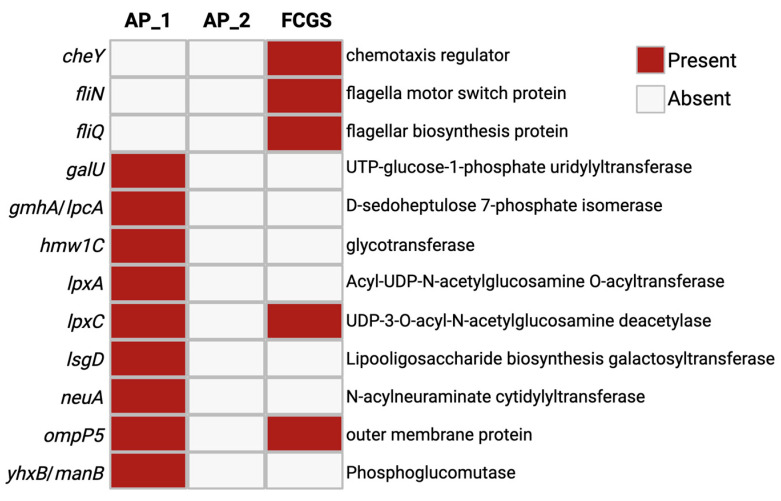
Expression of AMR and virulence factors differs between early AP and FCGS. Assembled transcripts from each community were run through CARD and VFDB with an identity cut-off of 80%. Data are displayed as presence or absence for each gene hit in each sample.

To assess whether changes in antimicrobial resistance gene (AMR) or virulence gene expression accompanied the noted microbial membership remodeling across time, the assembled transcripts for all three sampling points were examined with AMR and virulence factor databases (Figure 4). The AMR and virulence factor paralleled the observations of changing community composition across time, with AP_1 expressing primarily capsule remodeling factors, AP_2 with no significant hit, and FCGS expressing primarily motility-related genes. In AP_1, a total of nine different AMR and virulence-related genes were found in the metatranscriptome. These genes were primarily related to microbial membrane remodeling, and lipid and capsule production, including *galU*, *lpxA*, *lpxC*, and *neuA*. In FCGS there were five genes found, including *lpxC* and *ompP5*-like AP_1_. Unique to FCGS was the expression of the flagella-related genes *cheY*, *fliN*, and *fliQ*. Intriguingly, the AP_2 metatranscriptome had no identified transcripts related to AMR or virulence genes, even with a lower identity threshold of 80% that allows for sequence diversity between species. The expression of capsule and membrane-remodeling genes in the AP_1 microbiome suggests an active and ongoing response to environmental stressors while the flagella-related genes expressed in FCGS support the notion that oral microbes could be actively moving through the deteriorating tissues and binding oral structures.

**Figure 5:**
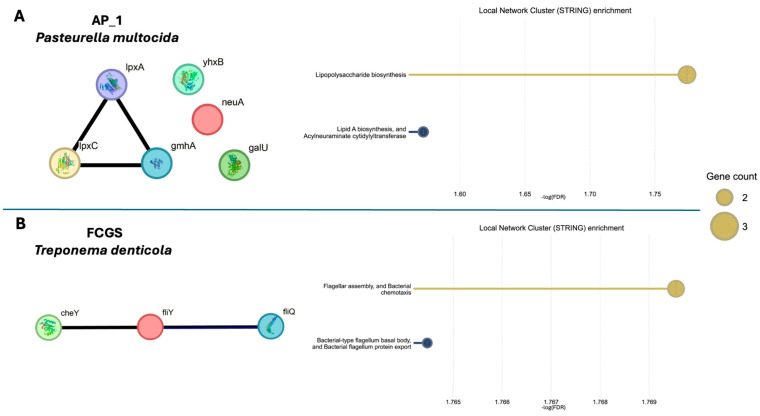
STRING enrichment of virulence factors supports functional differences between the microbiomes of AP_1 and FCGS. The STRING Database was used to perform a Local Network Cluster enrichment to determine functional profiles of the virulence factors found in each sample. One abundant organism in each sample was used as the genetic background for enrichment with (**A**) *Pasteurella multocida* used for AP_1 and (**B**) *Treponema denticola* for FCGS. AP_2 had no virulence factor hits and so was excluded from this analysis. Each circle labeled with the gene name represents a virulence factor with the connecting lines indicating gene cooccurrence, gene fusions, gene neighborhood, or experimentally determined protein-protein interactions.

Altered microbial activity, in concert with the observed taxonomic changes, may contribute to the progression of oral inflammation and disease. To illustrate the changing microbial activity between early AP and FCGS, the respective virulence factors found in each condition (Figure 4) were analyzed for protein-protein interactions, network cluster enrichment, and known protein associations using STRING (Figure 5). As abundant organisms in their respective microbiomes, *Pasteurella multocida* was used as the genetic background for the STRING enrichment analysis in AP_1,_ and *Treponema denticola* was used as the background for the FCGS STRING enrichment analysis. Membrane remodeling, from lipopolysaccharide biosynthesis (−log_10_FDR = 1.8) and lipid A biosynthesis and sialyation activity (−log_10_FDR = 1.6), was significantly enriched in the virulence factors found in AP_1. Contrastingly, the virulence factors found in the FCGS timepoint showed enrichment for the motility-related pathways of flagellar assembly and chemotaxis (−log_10_FDR = 1.8) and flagellum body and flagellum protein export (−log_10_FDR = 1.8). Though only covering the annotated virulence factors and thus narrow in scope, the notable difference between the type of enriched pathways in AP and FCGS suggests the microbiome alters microbial stress responses in ways unique to each disease state and community membership. Enrichment analysis of the AP_1 virulence factors suggests a need for increased stress tolerance in the microbiome through increased membrane construction and modification. Contrastingly, the virulence factors found in FCGS support motility in the microbiome and potentially indicate bacteria may be moving in the oral cavity or through the deteriorating dental structures.

## 4. Discussion

Shifts in the oral microbiome composition and function have been associated with the onset and progression of multiple periodontal diseases [21,40,41,42,43]. Considering this, diseases without an established etiology, like AP and FCGS, warrant a deeper investigation into potential microbial connections. Identifying microbial signatures of disease progression or potentially causative community structures is necessary for the development of effective treatments. The current lack of effective treatments makes AP and FCGS clinically challenging to manage for veterinarians and severely decreases the quality of life for patients [44]. Connecting the oral microbiome composition and function to disease status is therefore an important step in developing better clinical approaches to treating AP and FCGS.

AP and FCGS are treated as separate diseases of the oral cavity, but 7% of cats 2 years old or younger included in a retrospective study first diagnosed with AP went on to later develop FCGS [1]. The connection between these two diseases is not well understood, nor is the origin of either [1,8]. To that end, this case study provides insight into how the oral microbiome may link AP and FCGS in time and potentially how shifts in the microbial community can mark or contribute to disease progression. This case describes a single cat first diagnosed with AP and later with FCGS that was sampled with a caudal buccal swab at three time points encompassing two within the diagnosis of AP and one after the diagnosis of FCGS. This work combined longitudinal samples with progressing disease using total RNA sequencing revealed a dynamic shift in both microbiome composition and virulence-related activity.

The sampling of a single microbiome over time in a cat revealed a changing microbial community with markedly different compositions at the genus and species levels during progressive inflammatory disease in the oral cavity. Interestingly, while two of these microbiomes were sampled during the same diagnosis of AP, they were distinctly different in their composition. The first AP sample included species of *Capnocytophaga*, *Fusobacterium, Leptotrichia*, *Pasteurella*, and *Streptobacillus*. Contrastingly, the second sample of the AP-associated microbiome was dominated by *Aspergillus* and saw the rise of another fungal grouping *Kluveromyces*, along with increased bacterial membership of species from *Porphyromonas* and *Prevotella*. The microbiome community shifted once again in the FCGS sampling point, where *Treponema* and *Porphyromonas* were the two most dominant genera. This shifting profile both within the same diagnosis and to severe chronic disease highlights the complex and dynamic nature of the oral microbiome in the context of inflammatory disease progression.

The importance of considering the entire microbial community rather than just a few pathogens is further evidenced by the fact that, while *Porphyromonas* was present in both the AP and FCGS samples, its relative activity varied greatly between the two states. Similarly, *Treponema*, a genus associated with periodontal disease [45], only became dominant in FCGS. These findings suggest that the entire consortium of microbes, rather than a single or small group of “keystone” pathogens, plays a critical role in driving the transition to a dysbiotic state and the progression of inflammatory disease in the oral cavity [46]. Clinically, this indicates the need for a holistic consideration of the oral microbiome in treatment for inflammatory conditions, rather than a focus on a small set of pathogens [47,48].

Additionally important is the consideration of potential patient-specific differences that may influence the oral microbiome. Factors beyond disease status, like diet, age, and differing access to indoor or outdoor environments, can influence the composition of the oral microbiome and likewise contribute to disease status [20,49]. For instance, one study observed that cats fed primarily dry food had more microbial diversity than those fed wet food and additionally observed changes in microbiome composition between cohorts when their sample population was stratified by age [20]. Understanding the multiple factors that influence the oral microbiome composition is a necessary step in connecting microbiome composition and disease; however, it is also crucial to investigate in tandem the changing microbial function in the oral cavity.

In addition to profiling taxonomic shifts, the assembled transcripts from each microbiome were examined using a virulence factor database and an AMR database to reveal microbial functional changes associated with disease progression. The first sampled AP microbiome expressed genes related to membrane remodeling, including lipid synthesis and glycosylation. For instance, *galU* was found in the AP_1 sample and is a known contributor to virulence in multiple organisms through its role in modifying lipopolysaccharide (LPS) and connection to biofilm formation [50,51]. Relatedly, drivers of LPS component lipidA, *lpxA*, and *lpxC*, were also expressed in periodontitis. The expression of virulence factors primarily related to membrane modifications and subsequently to biofilm formation supports the ongoing hypothesis that biofilm activity contributes to the deterioration of oral tissues and structures as seen in periodontitis [52]. This observation of potential biofilm formation in AP is congruent with the notable microbial diversity observed in the AP microbiome. Previous work in human periodontitis revealed that increasing diversity was connected to progressing disease in part due to the expansion of biofilm niches in deepening gingival pockets [53,54]. While the samples in this case study were from the mucosa and not the subgingival compartment like the aforementioned human periodontal work, a parallel process of mucosal deterioration may be taking place in the cat’s oral tissue. Biofilms that contribute to worsening disease can be initiated by commensal oral organisms like *Streptococcus oralis*, which are early colonizers of the dental structure [55,56]. These early colonizers change the local environment, releasing metabolites and polysaccharide matrices that recruit other oral microbes, including periodontal pathogens like *T. denticola* and *P. gingivalis* [54,57]. This complex community structure on the surface of host structures then utilizes the host tissues as metabolic substrates and ultimately contributes to dental decay [52,54]. The manual disruption of oral biofilms in one study improved periodontal outcomes in humans with periodontitis and led to decreased diversity in the oral microbiome, further supporting that biofilms are contributing factors to dental decay [54]. Thus, the observation of diverse microbiomes and biofilm-related gene expression in AP supports the notion that microbial activity is contributing to worsening dental disease in cats. 

The expression of these biofilm and membrane-related virulence factors in the first AP microbiome is in contrast with the motility-related genes expressed by the FCGS-associated microbiome. *CheY*, *fliN*, and *fliQ* were all expressed in the FCGS microbiome and are involved in flagellar activity with *fliN* and *fliQ* contributing flagellar building blocks and *cheY* transmitting chemotaxis signals to direct movement [58]. The expression of such motility-related genes in the FCGS is an interesting observation in the context of previous work highlighting the role of flagella in instigating oral inflammation and systemic disease in host tissues [27,59,60,61]. The periplasmic flagella of oral pathogen *T. denticola* have been shown to initiate inflammation through the activation of the innate immune system via interaction with toll-like receptor 2 (TLR2) on host cells [62]. Other work in *T. denticola* has shown flagella to be important for bacterial penetration and thus successful infection of the host epithelium, while non-motile or chemotaxis-deficient counterparts were unable to invade host tissue [61]. The immunogenic nature of flagellar structure in conjunction with their importance for successful host colonization suggest the expression of these functions in the FCGS microbiome may be a notable finding from this study. FCGS results in the degradation of tissues and thus the release of host proteins and other compounds which may act as chemotaxis signals, inviting motile oral microbes to colonize the deteriorating host tissues and structures [63]. 

The exploration here of changing microbial composition and virulent activity in the oral microbiome of a cat with expanding oral inflammation provides multiple findings that can guide future work in this area. Though limited to a single cat as a case study, the results of this study suggest taxonomic differences are associated with progressing inflammation. Future work including a larger sampling population, longitudinal tracking of multiple cats, and experimental interventions will be necessary to determine whether these taxonomic and functional changes instigate or follow disease. Similarly, the identification of different microbiome virulence factor profiles in AP and FCGS supports that function, especially in connection to biofilm and motility, is an important facet when evaluating the connection between a shifting microbiome and host health. Collectively, this work provides a foundation for future work investigating how inflammatory diseases of the oral cavity are connected to the microbial consortia in the oral cavity.

## 5. Conclusions

Longitudinal sampling of the oral microbiome during progressing AP to the diagnosis of FCGS in a single cat revealed a dynamic microbiome with distinct profiles at the genus and species level. *Streptobacillus* species were most dominant in the first AP sample followed by a notable increase in *Aspergillus* at the second AP timepoint with *Porphyromonas* and *Treponema* as the predominant genera in the FCGS microbiome. In concert with the changing composition, the expression of virulence factors also changed through the progression of the disease. Genes primarily related to microbial membrane composition and modification were expressed in the first AP community, while motility-related virulence factors were the predominant virulence function in the FCGS profile. The longitudinal profiling of both the oral microbiome composition and virulence profile across time and in conjunction with progressive oral inflammation supports the microbiome in either responding to or inciting disease in the host. While further work is necessary to confirm these results at a population level, the observations here support that changing microbial composition and function mark disease progression from AP to FCGS and that the development of clinically relevant microbial markers for oral disease state is possible.

## Data Availability

Sequencing data generated and analyzed in this study can be found at the 100 K Pathogen Project on NCBI SRA under BioProject PRJNA1136879.
